# New records and an updated checklist of the herpetofauna from Tay Yen Tu Nature Reserve, north-eastern Vietnam

**DOI:** 10.3897/BDJ.13.e157862

**Published:** 2025-06-26

**Authors:** Anh Mai Luong, Tien Quang Phan, Truong Quang Nguyen, Tung Thanh Tran, Hien Thu Thi Ngo, Mona van Schingen-Khan, Thomas Ziegler, Cuong The Pham

**Affiliations:** 1 Institute of Biology, Vietnam Academy of Science and Technology, Hanoi, Vietnam Institute of Biology, Vietnam Academy of Science and Technology Hanoi Vietnam; 2 Graduate University of Science and Technology, Vietnam Academy of Science and Technology, Hanoi, Vietnam Graduate University of Science and Technology, Vietnam Academy of Science and Technology Hanoi Vietnam; 3 Vinh Phuc College, Vinh Phuc, Vietnam Vinh Phuc College Vinh Phuc Vietnam; 4 Department of Agriculture and Environment, Bac Giang, Vietnam Department of Agriculture and Environment Bac Giang Vietnam; 5 Federal Agency for Nature Conservation, CITES Scientific Authority, Bonn, Germany Federal Agency for Nature Conservation, CITES Scientific Authority Bonn Germany; 6 Cologne Zoo, Cologne, Germany Cologne Zoo Cologne Germany; 7 Institute of Zoology, University of Cologne, Cologne, Germany Institute of Zoology, University of Cologne Cologne Germany

**Keywords:** amphibians, Bac Giang Province, distribution, morphology, reptiles, taxonomy.

## Abstract

**Background:**

The Tay Yen Tu Nature Reserve is situated on the western side of the Yen Tu Mountain Range in Bac Giang Province, northern Vietnam. Since its establishment in 2002, several studies on the herpetofauna of this protected area have been carried out. For example, Nguyen et al. reported 25 species of snakes in 2002 and most recently, Hecht et al. reported 36 species of amphibians and 40 species of reptiles in the Nature Reserve in 2013. Remarkably, two new species of amphibians and two new species of reptiles have been discovered from Tay Yen Tu Nature Reserve since 2005.

**New information:**

As a result of our fieldwork in Tay Yen Tu Nature Reserve between 2015 and 2025, we recorded four species of amphibians and three species of reptiles for the first time from Bac Giang Province, comprising two species of *Leptobrachella*, one species of *Kaloula*, one species of *Hylarana*, one species of *Takydromus*, one species of *Sinomicrurus* and one species of *Xenopeltis*. We herein provide morphological data as well as ecological notes of the aforementioned species. In addition, we provide an updated list of 44 amphibian species and 49 reptile species recorded from Tay Yen Tu Nature Reserve.

## Introduction

The Tay Yen Tu Nature Reserve is located in Bac Giang Province, covering an area of 16,466 hectares of evergreen broad-leaved tropical lowland forest (Tordoff et al. 2004). In terms of herpetofaunal diversity, Nguyen et al. (2002) initially reported 25 species of snakes, while Hecht et al. (2013) recorded 76 species of amphibians and reptiles from Tay Yen Tu Nature Reserve. The Nature Reserve is also known as the type locality of four newly-discovered species, including *Tylototritonvietnamensis* (Böhme et al. 2005), *Odorranayentuensis* (Tran et al. 2008), *Opisthotropisvoquyi* (Ziegler et al. 2018) and *Achalinusemilyae* (Ziegler et al. 2019). In addition, three new country records of amphibians and reptiles were reported from the site, namely *Liuixalusfeii* Yang, Rao & Wang, 2015, *Odorranafengkaiensis* Wang, Lau, Yang, Chen, Liu, Pang & Liu, 2015 and *Occidozygashiwandashanensis* Chen, Peng, Liu, Huang, Liao & Mo, 2022 (Pham et al. 2018, Pham et al. 2020, Tran et al. 2023). Tay Yen Tu Nature Reserve is further home to the species that are on the brink of extinction, such as the Vulnerable *Tylototritonvietnamensis* or the Endangered *Shinisauruscrocodilurus* (van Schingen et al. 2016, Bernardes et al. 2017). However, natural habitats in the Nature Reserve are currently under threat, for example, by mining activities.

As a result of our fieldwork in Tay Yen Tu Nature Reserve between 2015 and 2025, we herein report seven new records of amphibians and reptiles for Tay Yen Tu Nature Reserve, as well as for Bac Giang Province.

## Materials and methods

**Sampling**

Field surveys were conducted by Pham T.C, An H.T., van Schingen-Khan M. and Barthel L. in May 2015; Pham C.T., Nguyen T.V., Phan T.Q. and Vu Q.M. in November 2024; and by Luong A.M., Pham C.T. and Phan T.Q. in March 2025 in Tay Yen Tu Nature Reserve (NR), Bac Giang Province. The coordinates (WGS 84) and elevations were determined by using a Garmin GPS 60CX device.

Field excursions and collection of specimens took place after sunset between 19:00 h and 23:00 h mainly along streams and ponds throughout the reserves. After taking photographs in life, specimens were anaesthetised and euthanised in a closed vessel with a piece of cotton wool containing ethyl acetate (Simmons 2002), fixed in 80% ethanol for five hours and then later transferred to 70% ethanol for permanent storage. Specimens referred to in this paper are deposited in the collection of the Institute of Biology (IB) (formerly known as the Institute of Ecology and Biological Resources, IEBR), Hanoi, Vietnam.

**Morphological examination**

For amphibians, measurements were taken on preserved specimens with a set of digital calipers for amphibian specimens to the nearest 0.1 mm. The following abbreviations were used: SVL = snout-vent length (from tip of snout to cloaca), HL = head length (measured as a parallel line to the vertebral column from posterior margin of mandible to tip of snout), HW = maximum head width (across angles of jaws), RL = rostral length (from anterior corner of orbit to tip of snout), NS = distance from nostril to the tip of snout, EN = distance from anterior corner of orbit to the nostril, TD = tympanum diameter, UAL = upper arm length (from axilla to elbow), FAL = forearm length (from elbow to tip of third finger), FL1–4 = Finger length I–IV, FeL = femur length (from vent to knee), TbL= tibia length (from knee to tarsus), TbW = maximum tibia width, FoL = foot length (from tarsus to the tip of fourth toe), TL1–5 = toe length I–V. For the webbing formula, we followed Glaw and Vences (2007). Sex was determined by the presence of nuptial pads and based on gonadal inspection.

For lizards and snakes, measurements were taken on preserved specimens with a measuring tape. Scalation of *Takydromus* was counted according to Wang et al. (2019). The number of ventral scales of snakes was counted according to Dowling (1951). The dorsal scale rows were given at one head length behind head, at mid-body and at one head length before vent, respectively. Scalation was studied by using a stereomicroscope (Leica M80). Bilateral scale counts were given as left/right. Identification of sex was made by inspection of presence of hemipenes.

## Taxon treatments

### Leptobrachella bourreti

(Dubois, 1983)

0A981298-0060-54DD-AD99-F4B2D00DC569

#### Materials

- **Type status:** Other material. **Occurrence:** catalogNumber: IB A.6374; individualCount: 1; sex: female; lifeStage: adult; occurrenceID: 7F4C9BA0-8609-531D-B1AC-7E14722D3659; **Taxon:** scientificNameID: *Leptobrachellabourreti*; scientificName: *Leptobrachellabourreti*; class: Amphibia; order: Anura; family: Megophryidae; genus: *Leptobrachella*; specificEpithet: *bourreti*; scientificNameAuthorship: Dubois, 1983; **Location:** country: Vietnam; countryCode: VN; stateProvince: Bac Giang; locality: Tay Yen Tu NR; verbatimElevation: 392 m; verbatimLatitude: 21°09.756’N; verbatimLongitude: 106°49.302’E; verbatimCoordinateSystem: WGS84; **Event:** eventDate: 18 May 2015; eventRemarks: collected by Pham T.C, van Schingen-Khan M., and Barthel L.; **Record Level:** language: en; collectionCode: Amphibia; basisOfRecord: PreservedSpecimen
- **Type status:** Other material. **Occurrence:** catalogNumber: IB A.6375; individualCount: 1; sex: female; lifeStage: adult; occurrenceID: A5624967-9981-529E-931B-56F7B5FFCC91; **Taxon:** scientificNameID: *Leptobrachellabourreti*; scientificName: *Leptobrachellabourreti*; class: Amphibia; order: Anura; family: Megophryidae; genus: *Leptobrachella*; specificEpithet: *bourreti*; scientificNameAuthorship: Dubois, 1983; **Location:** country: Vietnam; countryCode: VN; stateProvince: Bac Giang; locality: Tay Yen Tu NR; verbatimElevation: 476 m; verbatimLatitude: 21°09.835’N; verbatimLongitude: 106°48.638’E; verbatimCoordinateSystem: WGS84; **Event:** eventDate: 20 November 2024; eventRemarks: collected by Pham C.T., Nguyen T.V., Phan T.Q., and Vu Q.M; **Record Level:** language: en; collectionCode: Amphibia; basisOfRecord: PreservedSpecimen
- **Type status:** Other material. **Occurrence:** catalogNumber: IB A.6376; individualCount: 1; sex: male; lifeStage: adult; occurrenceID: C326FC76-7C0B-5CF9-86BD-397DF4AF40C6; **Taxon:** scientificNameID: *Leptobrachellabourreti*; scientificName: *Leptobrachellabourreti*; class: Amphibia; order: Anura; family: Megophryidae; genus: *Leptobrachella*; specificEpithet: *bourreti*; scientificNameAuthorship: Dubois, 1983; **Location:** country: Vietnam; countryCode: VN; stateProvince: Bac Giang; locality: Tay Yen Tu NR; verbatimElevation: 476 m; verbatimLatitude: 21°09.835’N; verbatimLongitude: 106°48.638’’E; verbatimCoordinateSystem: WGS84; **Event:** eventDate: 20 November 2024; eventRemarks: collected by Pham C.T., Nguyen T.V., Phan T.Q., and Vu Q.M; **Record Level:** language: en; collectionCode: Amphibia; basisOfRecord: PreservedSpecimen
- **Type status:** Other material. **Occurrence:** catalogNumber: IB A.6377; individualCount: 1; sex: male; lifeStage: adult; occurrenceID: BD1B4CEE-3741-515A-8681-BACE9AD73334; **Taxon:** scientificNameID: *Leptobrachellabourreti*; scientificName: *Leptobrachellabourreti*; class: Amphibia; order: Anura; family: Megophryidae; genus: *Leptobrachella*; specificEpithet: *bourreti*; scientificNameAuthorship: Dubois, 1983; **Location:** country: Vietnam; countryCode: VN; stateProvince: Bac Giang; locality: Tay Yen Tu NR; verbatimElevation: 347 m; verbatimLatitude: 21°09.837’N; verbatimLongitude: 106°49.314’E; verbatimCoordinateSystem: WGS84; **Event:** eventDate: 25 March 2025; eventRemarks: collected by Luong A.M., Pham C.T., and Phan T.Q.; **Record Level:** language: en; collectionCode: Amphibia; basisOfRecord: PreservedSpecimen

#### Description

**Figure 1.**
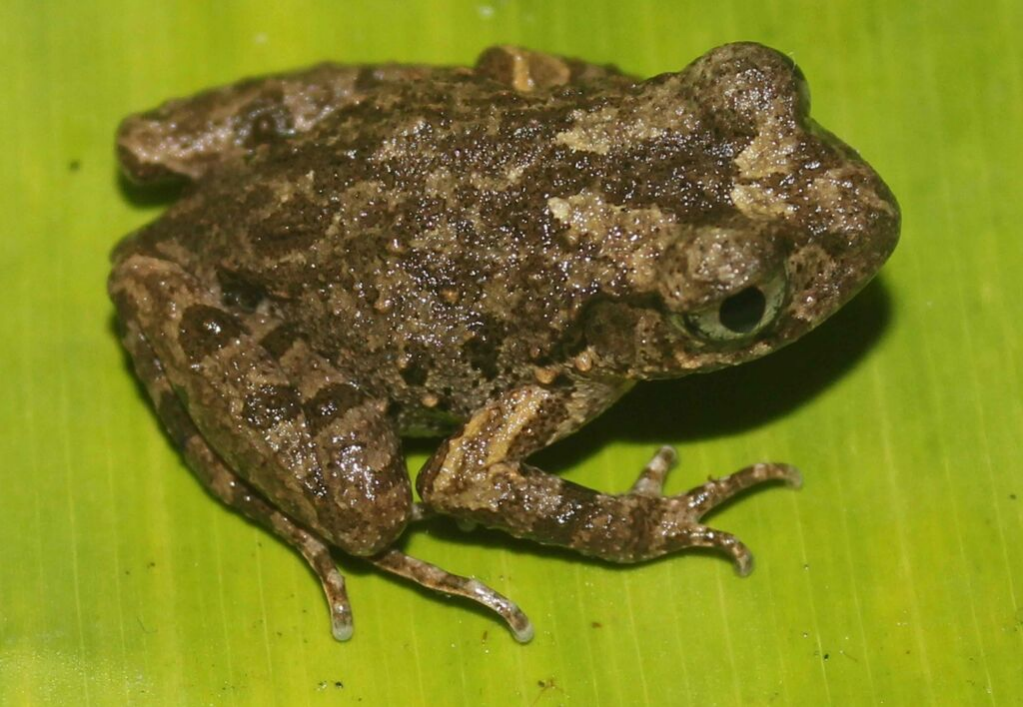
*Leptobrachellabourreti* (male, IB A.6373).

Morphological characters of the specimens from Tay Yen Tu NR agreed well with descriptions of Dubois (1983) and Ohler et al. (2011). Size small (SVL 25.7–26.2 mm in two males and SVL 32.0–33.6 mm in two females); head longer than wide (HL 10.4–10.6 mm, HW 8.9–9.2 mm in males and HL 12.5–12.9 mm, HW 11.1–11.6 in females); snout not protruding, longer than eye diameter (RL 4.0 mm, ED 3.4–3.7 mm in males and RL 3.7–5.1 mm, ED 3.8–4.6 mm in females); nostrils round, closer to the tip of snout than to eye (NS 1.6–1.9 mm, EN 2.3–2.5 mm in males and NS 1.6–1.9 mm, EN 2.9–3.2 mm in females); canthus rostralis round, loreal region slightly concave, obtuse; tympanum distinct; vomerine teeth absent; tongue notched posteriorly. Fore-limbs: Forearm slender, upper arm length (UAL 6.0–6.4 mm in males and UAL 7.2–8.2 mm in females), forearm length (FAL 13.6 mm in males and FAL 15.9–17.3 mm in females); relative finger lengths I < II < IV < III, tips of fingers enlarged; fingers free of webbing. Hind-limbs: Thigh slender (FeL 12.7–13.6 mm in males; FeL 14.2–16.1 mm in females); relative toe lengths I < II < V < III < IV; tips of toes not enlarged; webbing rudimentary; tibio-tarsal articulation reaching to the eye when leg adpressed along body. Skin: Dorsum smooth with some small flattened pustules; supratympanic fold distinct; throat, chest, belly and underside of limbs smooth. Colouration in life: Dorsal surface of head and body grey with dark marking; flank grey with black spots; dorsal surface of limbs grey with dark transverse bars; belly cream; throat and chest mottled with dark brown (Fig. 1).

#### Distribution

In Vietnam, this species has been recorded from Lao Cai, Lai Chau and Ha Giang Provinces (Nguyen et al. 2009, Frost 2025). Elsewhere, this species has been recorded from China and Thailand (Frost 2025).

#### Ecology

Specimens were found between 19:30 h and 20:30 h, on the ground or rocks in streams. The surrounding habitat was mixed secondary forest of medium and small hardwoods with bamboo and shrubs. Air temperatures at the sites ranged from 20.1°C to 25.7°C and relative humidity ranged 83% to 95%.

### Leptobrachella sungi

(Lathrop, Murphy, Orlov & Ho, 1998)

72557C68-EA1E-5E24-B60F-6B60DA55B6CC

#### Materials

- **Type status:** Other material. **Occurrence:** catalogNumber: IB A.6378; individualCount: 1; sex: female; lifeStage: adult; occurrenceID: DFF736DC-21D7-5BF2-B90E-5DC08EC34E0C; **Taxon:** scientificNameID: *Leptobrachellasungi*; scientificName: *Leptobrachellasungi*; class: Amphibia; order: Anura; family: Megophryidae; genus: *Leptobrachella*; specificEpithet: *sungi*; scientificNameAuthorship: Lathrop, Murphy, Orlov, and Ho, 1998; **Location:** country: Vietnam; countryCode: VN; stateProvince: Bac Giang; locality: Tay Yen Tu NR; verbatimElevation: 361 m; verbatimLatitude: 21°09.851’N; verbatimLongitude: 106°49.301’E; verbatimCoordinateSystem: WGS84; **Event:** eventDate: 16 May 2015; eventRemarks: collected by Pham C.T., van Schingen-Khan M., and Barthel L; **Record Level:** language: en; collectionCode: Amphibia; basisOfRecord: PreservedSpecimen
- **Type status:** Other material. **Occurrence:** catalogNumber: IB A.6379; individualCount: 1; sex: female; lifeStage: adult; occurrenceID: 4EA7B48E-42D8-5A18-9384-E44BD15ED7D0; **Taxon:** scientificNameID: *Leptobrachellasungi*; scientificName: *Leptobrachellasungi*; class: Amphibia; order: Anura; family: Megophryidae; genus: *Leptobrachella*; specificEpithet: *sungi*; scientificNameAuthorship: Lathrop, Murphy, Orlov, and Ho, 1998; **Location:** country: Vietnam; countryCode: VN; stateProvince: Bac Giang; locality: Tay Yen Tu NR; verbatimElevation: 400 m; verbatimLatitude: 21°09.662’N; verbatimLongitude: 106°49.236’E; verbatimCoordinateSystem: WGS84; **Event:** eventDate: 16 May 2015; eventRemarks: collected by Pham C.T., van Schingen-Khan M., and Barthel L.; **Record Level:** language: en; collectionCode: Amphibia; basisOfRecord: PreservedSpecimen

#### Description

**Figure 2.**
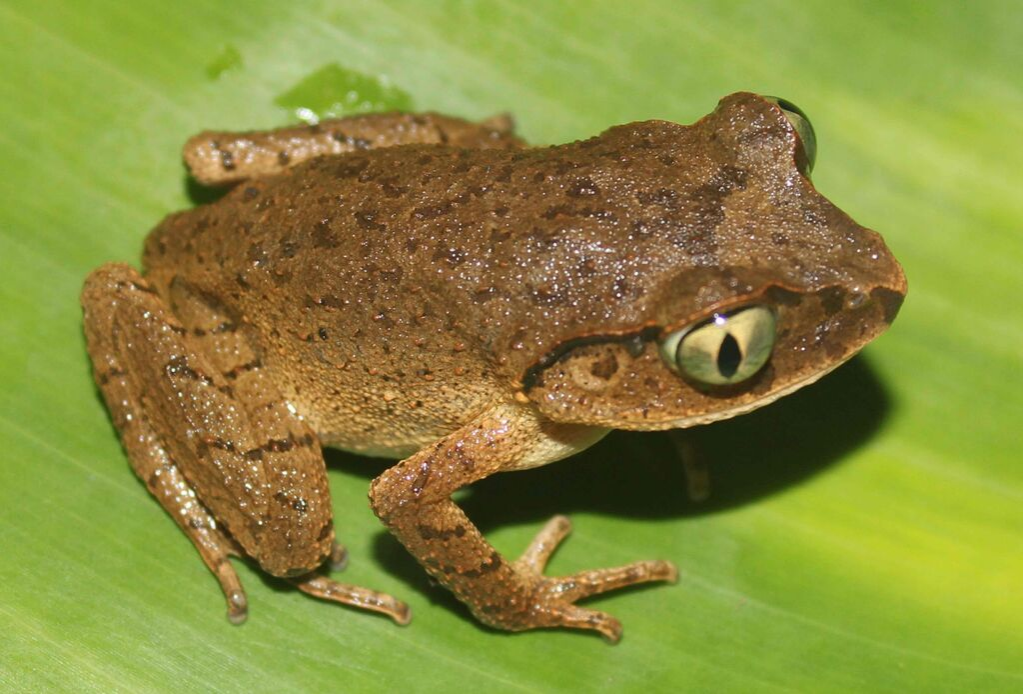
*Leptobrachellasungi* (female, IB A.6378).

Morphological characters of the specimens from Tay Yen Tu NR agreed well with descriptions of Lathrop et al. (1998) and Luong et al. (2019). Size medium (SVL 50.7–53.8 mm, n = 2); head longer than wide (HL 23.4–24.4 mm, HW 19.8–21.7 mm); snout round, longer than eye diameter (RL 8.0–9.0 mm, ED 6.8–6.9 mm); nostrils round, closer to the tip of snout than to eye (NS 3.0–4.3 mm, EN 5.1–5.8 mm); canthus rostralis distinct, loreal region concave; tympanum distinct; vomerine teeth absent; tongue notched posteriorly. Fore-limbs: Forearm slender, upper arm length (UAL 11.7–11.9 mm), forearm length (FAL 27.1–30.6 mm); relative finger lengths I < II < IV < III, tips of fingers not enlarged; fingers free of webbing. Hind-limbs: Thigh slender (FeL 22.0–25.8 mm); relative toe lengths I < II < V < III < IV; tips of toes not enlarged; webbing rudimentary. Skin: Dorsal surface of head and body, upper part of flanks with dispersed tubercles; upper eyelid granular; dorsolateral fold absent; ventral surface smooth. Colouration in life: Dorsal surface of head and body brown-copper with a dark marking between eyes; canthus and supratympanic fold brown; upper lip with brown bars; flanks light brown with dark points; dorsal surface of limbs with dark brown transverse bars; ventral surface opaque white (Fig. 2).

#### Distribution

In Vietnam, this species has been recorded from Vinh Phuc, Yen Bai, Lao Cai, Dien Bien, Phu Tho, Son La and Tuyen Quang Provinces (Nguyen et al. 2009, Luong et al. 2019, Frost 2025). Elsewhere, this species has been recorded from China (Frost 2025).

#### Ecology

Specimens were found between 19:30 h and 21:30 h, on the banks of rocky streams. The surrounding habitat was mixed secondary forest of medium and small hardwoods with bamboo and shrubs. Air temperatures at the sites ranged from 25.5°C to 32.8°C and relative humidity ranged from 63% to 82%.

### Kaloula pulchra

Gray, 1831

E21CDCCB-8B4F-55DA-BE1F-4D4C3626B93D

#### Materials

- **Type status:** Other material. **Occurrence:** catalogNumber: IB A.6380; individualCount: 1; sex: female; lifeStage: adult; occurrenceID: E5F34920-8355-5AD7-AADB-3E4DB3973D69; **Taxon:** scientificNameID: *Kaloulapulchra*; scientificName: *Kaloulapulchra*; class: Amphibia; order: Anura; family: Microhylidae; genus: *Kaloula*; specificEpithet: *pulchra*; scientificNameAuthorship: Gray, 1831; **Location:** country: Vietnam; countryCode: VN; stateProvince: Bac Giang; locality: Tay Yen Tu NR; verbatimElevation: 250 m; verbatimLatitude: 21°11.057’N; verbatimLongitude: 106°42.930’E; verbatimCoordinateSystem: WGS84; **Event:** eventDate: 4 July 2015; eventRemarks: collected by Pham T.C, van Schingen-Khan M., and Barthel L.; **Record Level:** language: en; collectionCode: Amphibia; basisOfRecord: PreservedSpecimen

#### Description

**Figure 3.**
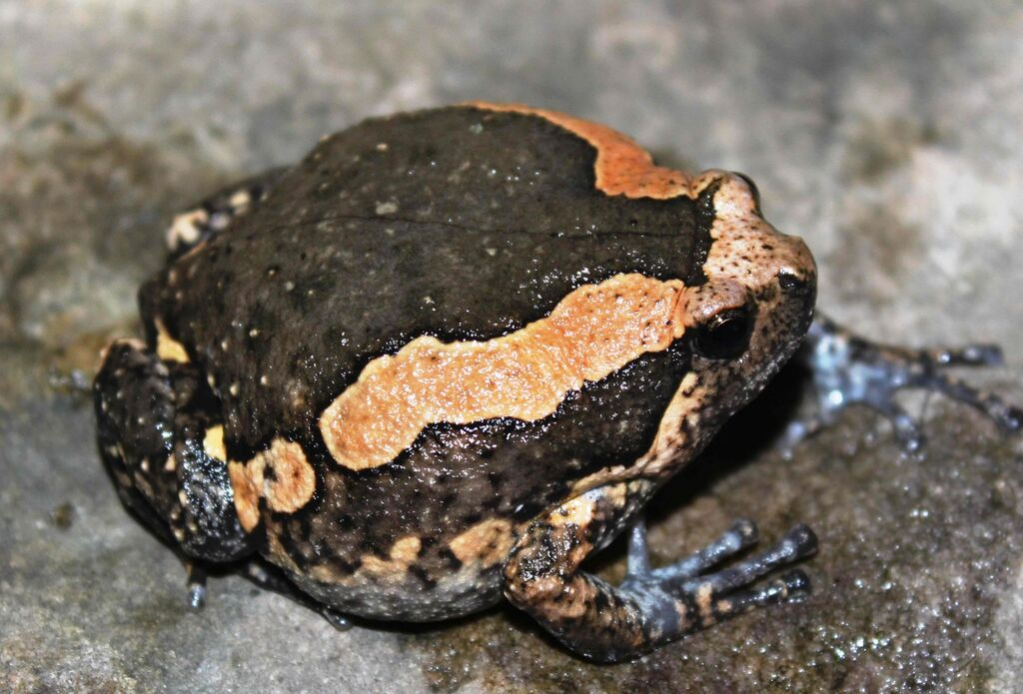
*Kaloulapulchra* (female, IB A.6380).

Morphological characters of the specimen from Tay Yen Tu NR agreed well with descriptions of Bourret (1942) and Taylor (1962). Size medium (SVL 60.4 mm); head shorter than wide (HL 17.4 mm, HW 20.6 mm); snout round, longer than eye diameter (RL 6.2 mm, ED 5.9 mm); nostrils round, closer to the tip of snout than to eye (NS 2.7 mm, EN 3.6 mm); canthus rostralis scarcely indicated, loreal region sloping obliquely, not concave; tympanum indistinct; vomerine teeth present; tongue notched posteriorly. Fore-limbs: Arms short; upper arm length (UAL 9.1 mm), forearm length (FAL 33.8 mm); relative finger lengths I < II < IV < III, tips of fingers enlarged; fingers free of webbing. Hind-limbs: Thigh short (FeL 24.9 mm); tibia three times longer than wide (TbL 22.5 mm, TbW 7.8 mm); relative toe lengths I < II < V < III < IV; tips of toes not enlarged; webbing rudimentary. Skin: Dorsal surface of head and body finely granular with some scattered pustular tubercles, ventral surface granulate. Colouration in life: Dorsal surface of head and body dark brown; an irregularly-edged light brown diagonal stripe from eye to groin; flanks dark brown; Dorsal surface of limbs with some light brown pattern; ventral surface light grey with grey markings (Fig. 3).

#### Distribution

In Vietnam, this species has been recorded from Bac Kan and Thai Nguyen Provinces in the north southwards to Tay Ninh and Ca Mau Provinces (Nguyen et al. 2009, Frost 2025). Elsewhere, this species has been recorded from Bangladesh, Cambodia, China, India, Indonesia, Laos, Myanmar, the Philippines, Singapore, Taiwan and Thailand (Frost 2025).

#### Ecology

The specimen was found at 21:00 h, on the forest path. The surrounding habitat was mixed secondary evergreen forest of medium and small hardwoods with shrubs and arrowroot. Air temperatures at the sites ranged from 26.3°C to 33.8°C and relative humidity ranged from 65% to 84%.

### Hylarana macrodactyla

Günther, 1858

A7FB3C9B-A934-544D-9AC2-44B12BD5AA1F

#### Materials

- **Type status:** Other material. **Occurrence:** catalogNumber: IB A.6381; individualCount: 1; sex: female; lifeStage: adult; occurrenceID: 128522C6-B4EE-5887-8476-46FE162BCE48; **Taxon:** scientificNameID: *Hylaranamacrodactyla*; scientificName: *Hylaranamacrodactyla*; class: Amphibia; order: Anura; family: Ranidae; genus: *Hylarana*; specificEpithet: *macrodactyla*; scientificNameAuthorship: Günther, 1858; **Location:** country: Vietnam; countryCode: VN; stateProvince: Bac Giang; locality: Tay Yen Tu NR; verbatimElevation: 330 m; verbatimLatitude: 21°10.131’N; verbatimLongitude: 106°48.727’E; verbatimCoordinateSystem: WGS84; **Event:** eventDate: 5 July 2015; eventRemarks: collected by Pham C.T.; **Record Level:** language: en; collectionCode: Amphibia; basisOfRecord: PreservedSpecimen

#### Description

**Figure 4.**
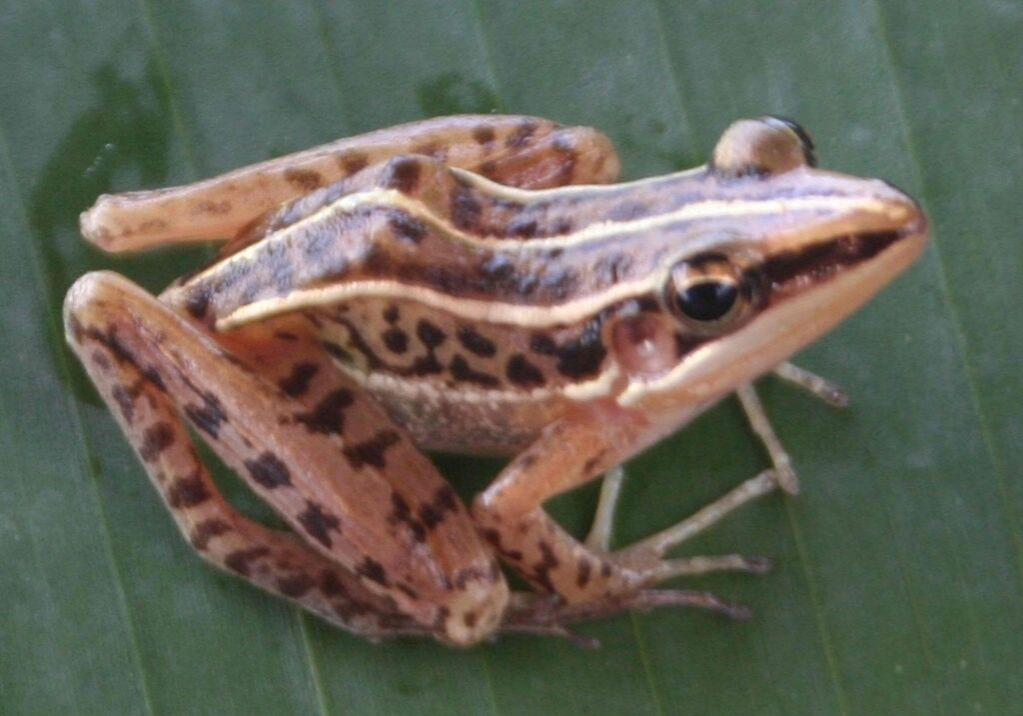
*Hylaranamacrodactyla* (female, IB A.6381).

Morphological characters of the specimen from Tay Yen Tu NR agreed well with descriptions of Bourret (1942) and Taylor (1962). Size medium (SVL 41.3 mm); head longer than wide (HL 15.8 mm, HW 9.9 mm); snout round, longer than eye diameter (RL 7.2 mm, ED 4.4 mm); nostrils round, closer to the tip of snout than to eye (NS 2.7 mm, EN 4.4 mm); canthus rostralis round, loreal region oblique, concave; tympanum distinct; vomerine teeth present; tongue notched posteriorly. Fore-limbs: Arms rather long, upper arm length (UAL 12.4 mm), hand length (FAL 35.4 mm); relative finger lengths I < II < IV < III, tips of fingers swollen; fingers free of webbing. Hind-limbs: Thigh slender (FeL 21.5 mm); tibia three times longer than wide (TbL 25.3 mm); relative toe lengths I < II < III < V < IV; tips of toes enlarged; toes about one-half webbed. Skin: Dorsal surface of head and body smooth, flank with few flat warts; median ventral and posterior surface of thigh with regular granulation or areolation; dorsolateral fold glandular. Colouration in life: Dorsal surface of head and body light brown with a narrow median light cream stripe, a cream stripe extending from eye to groin, along dorsolateral fold; flank with small brownish spots, upper limbs light brown with transverse cream bars; ventral surface cream (Fig. 4).

#### Distribution

In Vietnam, this species has been recorded from Ha Giang and Yen Bai Provinces in the north southwards to Ho Chi Minh and Ca Mau Provinces (Nguyen et al. 2009, Frost 2025). Elsewhere, this species has been recorded from Cambodia, China, Laos, Malaysia, Myanmar and Thailand (Nguyen et al. 2009, Frost 2025).

#### Ecology

The specimen was found at 20:30 h in a small stream. The surrounding habitat was mixed secondary evergreen forest of medium and small hardwoods with shrubs and arrowroot. Air temperatures at the sites ranged from 27.5°C to 34.1°C and relative humidity ranged from 68% to 82%.

### Takydromus sexlineatus

Daudin, 1802

4D24DDE4-A2D8-5018-B1F8-AEB8628DE982

#### Materials

- **Type status:** Other material. **Occurrence:** catalogNumber: IB 3664; individualCount: 1; sex: female; lifeStage: adult; occurrenceID: 5700017B-A9B8-5A6C-BB46-5D3C4C4AC4F0; **Taxon:** scientificNameID: *Takydromussexlineatus*; scientificName: *Takydromussexlineatus*; class: Reptile; order: Squamata; family: Lacertidae; genus: *Takydromus*; specificEpithet: *sexlineatus*; scientificNameAuthorship: Daudin, 1802; **Location:** country: Vietnam; countryCode: VN; stateProvince: Bac Giang; locality: Tay Yen Tu NR; verbatimElevation: 265 m; verbatimLatitude: 21°10.940’N; verbatimLongitude: 106°42.725’E; verbatimCoordinateSystem: WGS84; **Event:** eventDate: 2 July 2015; eventRemarks: collected by Pham C.T.; **Record Level:** language: en; collectionCode: Reptile; basisOfRecord: PreservedSpecimen

#### Description

**Figure 5.**
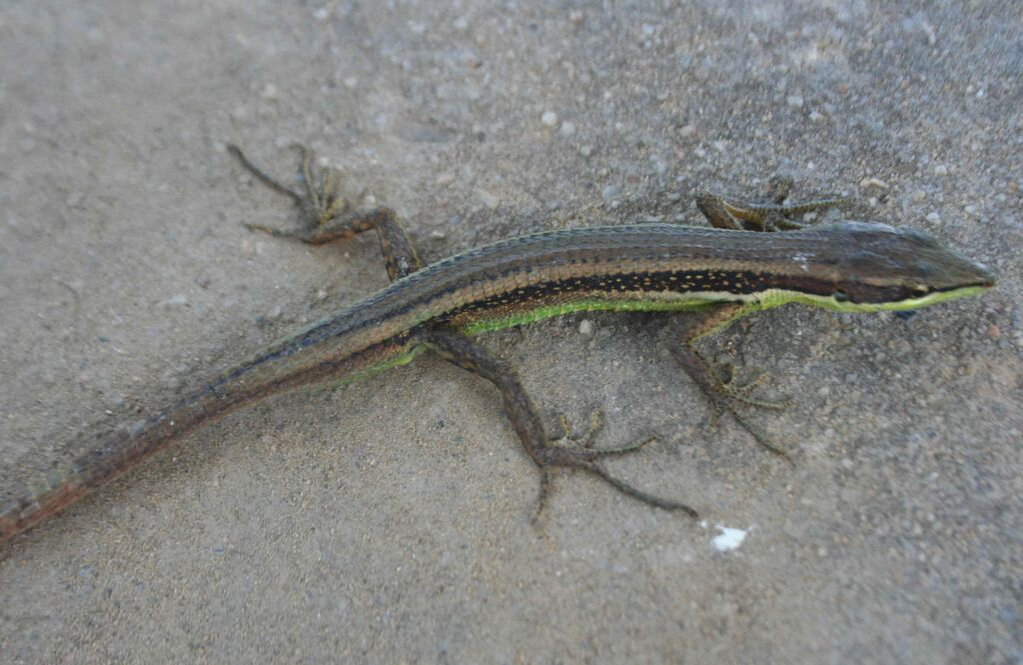
*Takydromussexlineatus* (female, IB 3664).

Morphological characters of the specimen from Tay Yen Tu NR agreed well with descriptions of Taylor (1963), Ziegler et al. (1999) and Nguyen et al. (2018). Body slender with an extra-long tail (SVL 52.2 mm, TaL 38.5 mm, tail loss); head longer than wide; horizontal diameter of orbit longer than distance from anterior corner of orbit to nostril; supralabials 7/7; infralabials 5/5; tympanum exposed, horizontal diameter (1.8 mm) less than that of the orbit; chin shields in three pairs; dorsal scales enlarged and keeled in six rows at mid-body, without a non-contiguous vertebral row of smaller scales; ventral scales widened, in six rows at mid-body; lateral scales in 12 rows at mid-body on each sides, smaller in size than dorsal and ventral scales; femoral pores four on each side; subdigital lamellae broadened. Colouration in life: Dorsal surface of head and body brown with some small black spots; upper part of flanks black with numerous yellowish spots, lower part of flanks light brown-green; dorsal surface of limbs and tail base with black spots; ventral surface whitish-cream (Fig. 5).

#### Distribution

In Vietnam, this species has been recorded from Lao Cai and Ha Giang Provinces in the north southwards to Tay Ninh and Binh Duong Provinces (Nguyen et al. 2009, Uetz et al. 2025). Elsewhere, this species has been recorded from Cambodia, China, India, Indonesia, Laos, Malaysia, Myanmar and Thailand (Nguyen et al. 2009, Uetz et al. 2025).

#### Ecology

The specimen was found at 19:30 h, on the forest path. The surrounding habitat was mixed secondary evergreen forest of medium and small hardwoods with bamboo, shrubs and arrowroot. Air temperatures at the sites ranged from 26.3°C to 33.8°C and relative humidity ranged from 65% to 84%.

### Sinomicrurus macclellandi

(Reinhardt, 1844)

D76CED28-16A1-5AA7-A808-A0BAEBD6D1ED

#### Materials

- **Type status:** Other material. **Occurrence:** catalogNumber: IB R.6384; individualCount: 1; sex: female; lifeStage: adult; occurrenceID: B856FE9B-795D-5DAE-B583-12491C9939EF; **Taxon:** scientificNameID: *Sinomicrurusmacclellandi*; scientificName: *Sinomicrurusmacclellandi*; class: Reptile; order: Squamata; family: Elapidae; genus: *Sinomicrurus*; specificEpithet: *macclellandi*; scientificNameAuthorship: Reinhardt, 1844; **Location:** country: Vietnam; countryCode: VN; stateProvince: Bac Giang; locality: Tay Yen Tu NR; verbatimElevation: 288 m; verbatimLatitude: 21°10.507’N; verbatimLongitude: 105°43.337’E; verbatimCoordinateSystem: WGS84; **Event:** eventDate: 2 July 2015; eventRemarks: collected by Pham C.T.; **Record Level:** language: en; collectionCode: Reptile; basisOfRecord: PreservedSpecimen

#### Description

**Figure 6.**
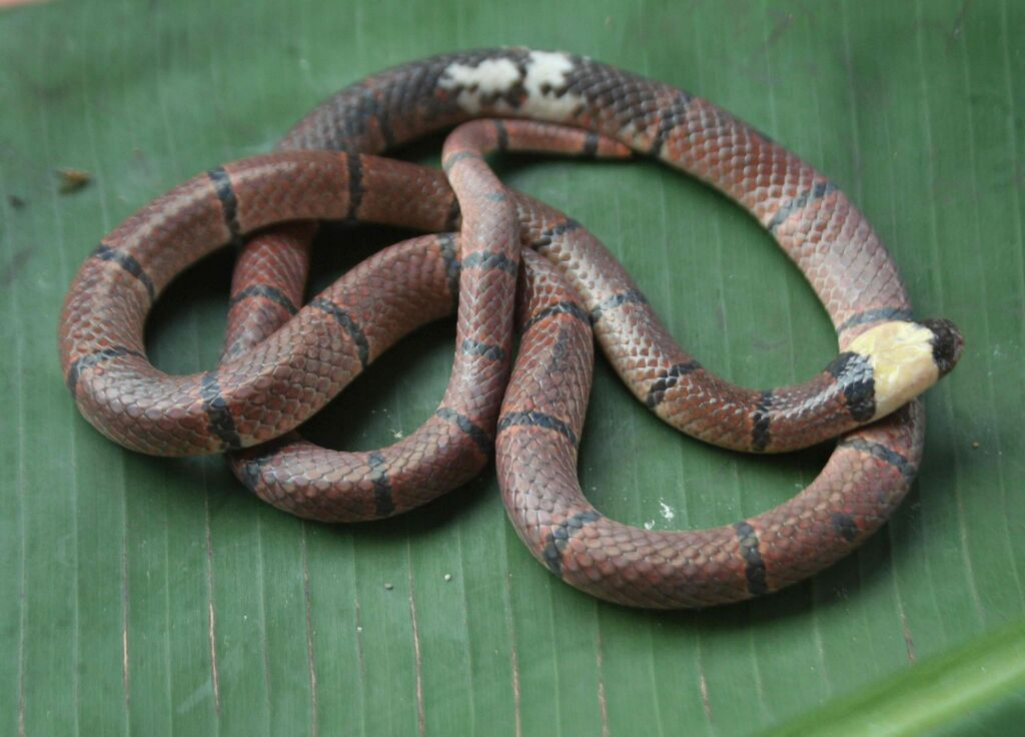
*Sinomicrurusmacclellandi* (female, IB A.6384).

Morphological characters of the specimen from Tay Yen Tu NR agreed well with descriptions of Pope (1935), Taylor (1965) and Luu et al. (2020). SVL 472.5 mm, TaL 57.61 mm; head not distinct from neck; eye small, pupil round; internasals divided; rostral as wide as high, clearly visible from above; frontal longer than wide, but shorter than parietals; loreal absent; preocular 1/1; postoculars 2/2, bordering anterior temporal on each side; anterior temporal 1/1, posterior temporals 1+2; supralabials 7/7, third to fourth touching the eye, sixth largest; infralabials 6/6, first to fourth bordering chin shields; chin shields in two pairs, in contact medially; dorsal scale rows 13–13–13, smooth; ventrals 206; cloacal shield undivided; subcaudals 32, divided; tail with pointed tip. Colouration in life: Dorsal reddish brown, with 28 black cross-bands and three on tail; head black with a wide white cross-band behind eyes; ventral surface cream with black bands and black squarish marks (Fig. 6).

#### Distribution

In Vietnam, this species has been recorded from Lao Cai and Cao Bang Provinces in the north southwards to Lam Dong and Dong Nai Provinces (Nguyen et al. 2009, Uetz et al. 2025). Elsewhere, this species has been recorded from Bangladesh, Bhutan, China, India, Japan, Laos, Myanmar, Nepal, Taiwan and Thailand (Nguyen et al. 2009, Uetz et al. 2025).

#### Ecology

The specimen was found at 21:00 h, on the bank of a rocky stream. The surrounding habitat was mixed secondary evergreen forest of medium and small hardwoods with shrubs and arrowroot. Air temperatures at the sites ranged from 25.3°C to 33.8°C and relative humidity ranged from 65% to 84%.

### Xenopeltis hainanensis

Hu & Zhao, 1972

AB979C79-04D7-5430-8A35-F4189066897C

#### Materials

- **Type status:** Other material. **Occurrence:** catalogNumber: IB R.6385; individualCount: 1; sex: male; lifeStage: adult; occurrenceID: 7C03A3F4-2C8D-598E-854E-9136EA4B8032; **Taxon:** scientificNameID: *Xenopeltishainanensis*; scientificName: *Xenopeltishainanensis*; class: Reptile; order: Squamata; family: Xenopeltidae; genus: *Xenopeltis*; specificEpithet: *hainanensis*; scientificNameAuthorship: Hu & Zhao, 1972; **Location:** country: Vietnam; countryCode: VN; stateProvince: Bac Giang; locality: Tay Yen Tu NR; verbatimElevation: 360 m; verbatimLatitude: 21°10.575’N; verbatimLongitude: 106°42.623’E; verbatimCoordinateSystem: WGS84; **Event:** eventDate: 1 July 2015; eventRemarks: collected by Pham C.T.; **Record Level:** language: en; collectionCode: Reptile; basisOfRecord: PreservedSpecimen

#### Description

**Figure 7.**
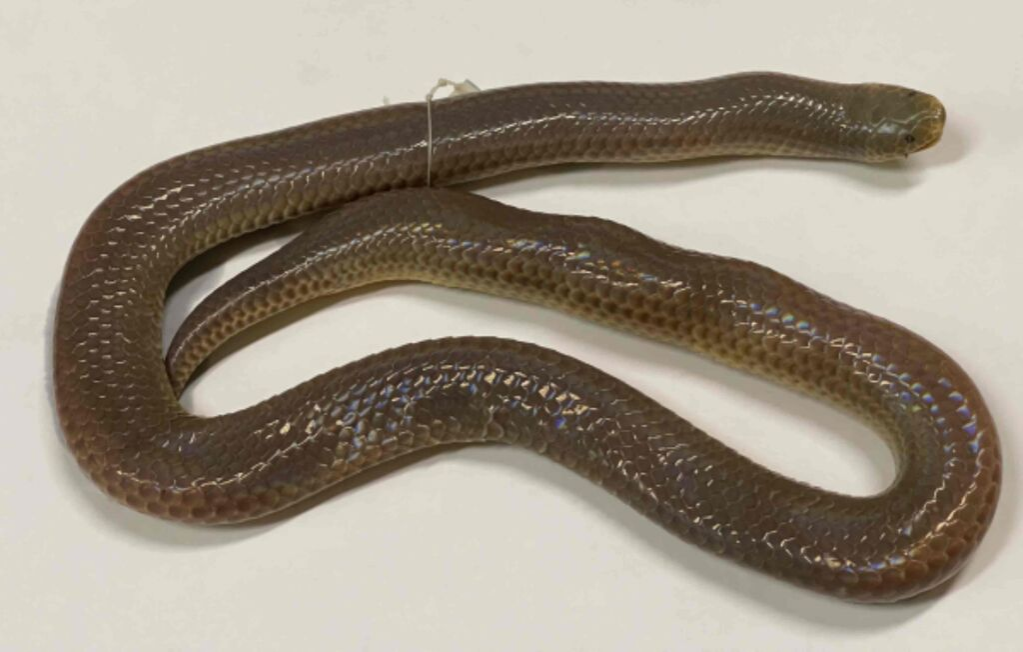
*Xenopeltishainanensis* (male, IB A.6385).

Morphological characters of the specimen from Tay Yen Tu NR agreed well with descriptions of Kizirian et al. (2003) and Orlov et al. (2022). SVL 586.7 mm, TaL 46.1 mm; head not distinct from neck; eye small, pupil round; internasals divided; rostral as wide as high, clearly visible from above; frontal longer than wide, but shorter than parietals; loreal absent; preocular 1/1; postoculars 2/2, bodering anterior temporal on each side; anterior temporal 1/1, posterior temporals 2; supralabials 7/7, third and fourth touching the eye, sixth largest; infralabials 7/7, first to third bordering chin shields; chin shields in two pairs, in contact medially; dorsal scale rows 15–15–15, smooth; ventrals 163; cloacal shield divided; subcaudals 19, divided; tail with pointed tip. Colouration in life: Dorsal uniform stone grey; upper lip, throat and ventral surface opaque white (Fig. 7).

#### Distribution

In Vietnam, this species has been recorded from Yen Bai, Cao Bang, Lang Son, Vinh Phuc, Hai Duong, Ha Tinh and Quang Binh Provinces (Nguyen et al. 2009, Uetz et al. 2025). Elsewhere, this species has been recorded from China (Nguyen et al. 2009, Uetz et al. 2025).

#### Ecology

The specimen was found at 22:00 h, on the bank of a rocky stream. The surrounding habitat was mixed secondary evergreen forest of medium and small hardwoods with bamboo, shrubs and arrowroot. Air temperatures at the sites ranged from 25.2°C to 33.6°C and relative humidity ranged from 67% to 85%.

## Checklists

### An updated checklist of the herpetofauna of Tay Yen Tu Nature Reserve, Bac Giang Province

#### Amphibia

D01FA0AC-2AD1-5214-86C9-5826E4CF27C3

#### Anura

62BDC46B-2D61-59BA-A74A-935F104765C7

#### Bufonidae

C82E4E1A-D732-5417-8411-F93776F28113

#### Duttaphrynus melanostictus

(Schneider, 1799)

F4150039-3E54-5FF7-8650-4B69746AA96B

##### Distribution

Vietnam, Sri Lanka, India, Nepal, China, Myanmar, Laos, Thailand, Cambodia, Malaysia, the Philippines.

##### Notes

Distribution in Tay Yen Tu Nature Reserve, Bac Giang Province (Hecht et al. 2013).

#### Ingerophrynus galeatus

(Günther, 1864)

79EC923D-9E99-5F23-8536-09D3908B7C6D

##### Distribution

Vietnam, China, Laos, Cambodia.

##### Notes

Distribution in Tay Yen Tu Nature Reserve, Bac Giang Province (Hecht et al. 2013).

#### Megophryidae

97557446-1C9B-507E-A014-D40D2647B1D7

#### Leptobrachella bourreti

(Dubois, 1983)

2EB80932-B08A-56E2-82DC-B7B1E183B808

##### Distribution

Vietnam, Laos, Thailand.

##### Notes

First record for Bac Giang Province.

#### Leptobrachella nyx

(Ohler, Wollenberg, Grosjean, Hendrix, Vences, Ziegler & Dubois, 2011)

079D5DA4-5AC2-57A9-B26F-8CA733CA90ED

##### Distribution

Vietnam, China.

##### Notes

Distribution in Tay Yen Tu Nature Reserve, Bac Giang Province (Hecht et al. 2013 and Chen et al. 2018).

#### Leptobrachella sungi

(Lathrop, Murphy, Orlov & Ho, 1998)

6F464B0B-D7D8-5037-BA49-5EB4A7D89050

##### Distribution

Vietnam, China.

##### Notes

First record for Bac Giang Province.

#### Leptobrachium chapaense

(Bourret, 1937)

26F239AC-B441-57A3-A245-8B2057F47CDE

##### Distribution

Vietnam, China, Myanmar, Laos, Thailand.

##### Notes

Distribution in Tay Yen Tu Nature Reserve, Bac Giang Province (Hecht et al. 2013).

#### Ophryophryne microstoma

Boulenger, 1903

0D744C2B-2339-5CF9-92F9-8C8ECE81515B

##### Distribution

Vietnam, China, Laos, Thailand, Cambodia.

##### Notes

Distribution in Tay Yen Tu Nature Reserve, Bac Giang Province (Hecht et al. 2013).

#### Xenophrys maosonensis

(Bourret, 1937)

E02772DC-4AAC-5E14-BFFD-32B66D4F1453

##### Distribution

Vietnam, China.

##### Notes

Distribution in Tay Yen Tu Nature Reserve, Bac Giang Province (Hecht et al. 2013 and Mahony et al. 2018).

#### Microhylidae

5CF0153F-DF4E-59D9-9ECD-199FF0E99587

#### Kalophrynus interlineatus

(Blyth, 1855)

72489D70-2A46-5513-ADA4-CBFE450D7271

##### Distribution

Vietnam, India, China, Myanmar, Laos, Thailand, Cambodia, Indonesia.

##### Notes

Distribution in Tay Yen Tu Nature Reserve, Bac Giang Province (Hecht et al. 2013).

#### Kaloula pulchra

Gray, 1831

E400D7E9-7179-5989-8038-D449467030D4

##### Distribution

Vietnam, India, Bangladesh, China, Myanmar, Laos, Thailand, Cambodia, Indonesia.

##### Notes

First record for Bac Giang Province.

#### Microhyla butleri

Boulenger, 1900

6AA81BF4-8507-5EC8-AE53-B4FDCF501D03

##### Distribution

Vietnam, China, Myanmar, Laos, Thailand, Cambodia, Malaysia, Singapore.

##### Notes

Distribution in Tay Yen Tu Nature Reserve, Bac Giang Province (Hecht et al. 2013).

#### Microhyla heymonsi

Vogt, 1911

3E7042D1-C713-52ED-9A21-291B9786BEDB

##### Distribution

Vietnam, India, China, Laos, Thailand, Cambodia, Malaysia, Indonesia.

##### Notes

Distribution in Tay Yen Tu Nature Reserve, Bac Giang Province (Hecht et al. 2013).

#### Microhyla pulchra

(Hallowell, 1861)

B1178C6B-4BB3-57E4-89FA-D98A5A305E72

##### Distribution

Vietnam, India, China, Laos, Thailand, Cambodia.

##### Notes

Distribution in Tay Yen Tu Nature Reserve, Bac Giang Province (Hecht et al. 2013).

#### Dicroglossidae

0ACAA36A-D614-5B99-8EDC-996AB8FBC6D5

#### Fejervarya limnocharis

(Gravenhorst, 1829)

B03B9124-A9BD-53AD-95DE-50636F7AF0EB

##### Distribution

Vietnam, Afghanistan, Pakistan, India, Nepal, Sri Lanka, Bangladesh, China, Myanmar, Laos, Thailand, Cambodia, Malaysia, Singapore, Indonesia, the Philippines, Japan.

##### Notes

Distribution in Tay Yen Tu Nature Reserve, Bac Giang Province (Nguyen et al. 2009).

#### Hoplobatrachus chinensis

(Osbeck, 1765)

ABBCB67D-E372-542D-8637-3089720357BE

##### Distribution

Vietnam, Myanmar, China, Cambodia, Laos, Thailand, Malaysia, Borneo, the Philippines.

##### Notes

Distribution in Tay Yen Tu Nature Reserve, Bac Giang Province (Hecht et al. 2013).

#### Limnonectes bannaensis

Ye, Fei, Xie & Jiang, 2007

B66D6192-3641-5FD4-A9C2-4B8B9EE7F1F0

##### Distribution

China, Thailand, Myanmar, Laos, Vietnam.

##### Notes

Distribution in Tay Yen Tu Nature Reserve, Bac Giang Province (Hecht et al. 2013).

#### Limnonectes quangninhensis

Pham, Le, Nguyen, Ziegler, Wu & Nguyen, 2017

6A22B8B6-3A10-5CED-BD63-BCA228AD6BC0

##### Distribution

Vietnam.

##### Notes

Distribution in Tay Yen Tu Nature Reserve, Bac Giang Province (Pham et al. 2019).

#### Quasipaa acanthophora

Dubois & Ohler, 2009

CE030A54-C30E-5F56-B6E5-C1C534D9D488

##### Distribution

Vietnam.

##### Notes

Distribution in Tay Yen Tu Nature Reserve, Bac Giang Province (Hecht et al. 2013).

#### Occidozyga martensii

(Peters, 1867)

F3E94963-5FAD-5A86-BE7F-D8CCDD632C13

##### Distribution

Vietnam, China, Laos, Thailand, Cambodia.

##### Notes

Distribution in Tay Yen Tu Nature Reserve, Bac Giang Province (Hecht et al. 2013).

#### Occidozyga shiwandashanensis

Chen, Peng, Liu, Huang, Liao & Mo, 2022

88D6E11F-C7F7-52D6-B016-7E162F4E7CA6

##### Distribution

Vietnam, China.

##### Notes

Distribution in Tay Yen Tu Nature Reserve, Bac Giang Province (Tran et al. 2023).

#### Ranidae

4CE50761-B5A5-5EB0-9EEC-37E091258275

#### Amolops shihaitaoi

Wang, Li, Du, Hou & Yu, 2022

B8F5A5B8-6183-55C9-8696-F0E308DA0E53

##### Distribution

Vietnam, China.

##### Notes

Distribution in Tay Yen Tu Nature Reserve, Bac Giang Province (Hecht et al. 2013 and Wang et al. 2022).

#### Nidirana chapaensis

(Bourret, 1937)

117D5F87-85B8-5497-AC34-453347BF1A00

##### Distribution

Vietnam, Laos, China.

##### Notes

Distribution in Tay Yen Tu Nature Reserve, Bac Giang Province (Hecht et al. 2013).

#### Hylarana guentheri

(Boulenger, 1882)

42738D78-9228-56C5-8A99-CADD89590137

##### Distribution

Vietnam, China, Myanmar, Laos.

##### Notes

Distribution in Tay Yen Tu Nature Reserve, Bac Giang Province (Hecht et al. 2013).

#### Hylarana maosonensis

Bourret, 1937

DC2DCC8D-9B5A-5271-A1A5-CF065B1118E3

##### Distribution

Vietnam, Laos.

##### Notes

Distribution in Tay Yen Tu Nature Reserve, Bac Giang Province (Hecht et al. 2013).

#### Hylarana macrodactyla

(Günther, 1858)

D682353A-F892-51AA-B9DC-6CCECA9381EA

##### Distribution

Vietnam, China, Myanmar, Laos, Thailand, Cambodia, Malaysia.

##### Notes

First record for Bac Giang Province.

#### Hylarana annamitica

(Sheridan & Stuart, 2018)

37C51FFE-F6FE-5490-82C1-C42CB241EB7B

##### Distribution

Vietnam, Laos.

##### Notes

Distribution in Tay Yen Tu Nature Reserve, Bac Giang Province (Hecht et al. 2013 and Sheridan and Stuart 2018).

#### Hylarana taipehensis

(Van Denburgh, 1909)

09B71E68-EF95-5AAD-B3B2-59553D8D0576

##### Distribution

Vietnam, India, Nepal, Bangladesh, China, Myanmar, Laos, Thailand, Cambodia.

##### Notes

Distribution in Tay Yen Tu Nature Reserve, Bac Giang Province (Hecht et al. 2013).

#### Odorrana fengkaiensis

Wang, Lau, Yang, Chen, Liu, Pang & Liu, 2015

E0D600BC-102C-51C7-A936-F3ABA6DA608E

##### Distribution

Vietnam, China.

##### Notes

Distribution in Tay Yen Tu Nature Reserve, Bac Giang Province (Hecht et al. 2013 and Pham et al. 2020).

#### Odorrana graminea

(Boulenger, 1900)

02FBE536-09E7-5754-9247-53B59D03AB4C

##### Distribution

Vietnam, China.

##### Notes

Distribution in Tay Yen Tu Nature Reserve, Bac Giang Province (Hecht et al. 2013).

#### Odorrana trankieni

(Orlov, Le & Ho 2003)

09FDDA89-16B4-5454-A8D8-C1FD7A151025

##### Distribution

China, Vietnam.

##### Notes

Distribution in Tay Yen Tu Nature Reserve, Bac Giang Province (Hecht et al. 2013 and Pham et al. 2020).

#### Odorrana yentuensis

Tran, Orlov & Nguyen, 2008

BAC3C193-96A7-5CD8-8BDA-AF41E1B4D714

##### Distribution

China, Vietnam.

##### Notes

Distribution in Tay Yen Tu Nature Reserve, Bac Giang Province (Hecht et al. 2013).

#### Rana johnsi

Smith, 1921

D1C0BE12-91A6-55FB-939A-56E1301C17FF

##### Distribution

Vietnam, China, Laos, Thailand, Cambodia.

##### Notes

Distribution in Tay Yen Tu Nature Reserve, Bac Giang Province (Hecht et al. 2013).

#### Rhacophoridae

90CDDD18-85D5-5D51-BE81-171BE15D215C

#### Feihyla vittata

(Boulenger, 1887)

0C9DC013-043D-59B4-A280-A632F88CE7B6

##### Distribution

Vietnam, India, China, Myanmar, Laos, Thailand, Cambodia.

##### Notes

Distribution in Tay Yen Tu Nature Reserve, Bac Giang Province (Hecht et al. 2013).

#### Kurixalus bisacculus

(Taylor, 1962)

D1D0D3C7-0F01-5615-81CF-C83CD775CCBE

##### Distribution

Thailand, Cambodia, Laos, Myanmar, China, Vietnam.

##### Notes

Distribution in Tay Yen Tu Nature Reserve, Bac Giang Province (Hecht et al. 2013).

#### Liuixalus feii

Yang, Rao & Wang, 2015

42029D5F-E308-5894-A998-AE75914CCBBE

##### Distribution

China, Vietnam.

##### Notes

Distribution in Tay Yen Tu Nature Reserve, Bac Giang Province (Pham et al. 2018).

#### Polypedates megacephalus

Hallowell, 1861

81DD0519-ED45-5BBF-B27E-8A8899FADEDC

##### Distribution

Vietnam, India, China, Myanmar, Taiwan, Laos, Thailand, Japan.

##### Notes

Distribution in Tay Yen Tu Nature Reserve, Bac Giang Province (Hecht et al. 2013).

#### Polypedates mutus

(Smith, 1940)

41B2B311-DF56-5B14-84BE-19DB1E5CFAE4

##### Distribution

Vietnam, China, Myanmar, Laos, Thailand.

##### Notes

Distribution in Tay Yen Tu Nature Reserve, Bac Giang Province (Hecht et al. 2013).

#### Rhacophorus napoensis

Li, Liu, Yu & Sun, 2022

4CEFB001-BA78-543C-A81D-DCE0479C186F

##### Distribution

Vietnam, China.

##### Notes

Distribution in Tay Yen Tu Nature Reserve, Bac Giang Province (Hecht et al. 2013, Li et al. 2022 and Nguyen et al. 2024).

#### Theloderma albopunctatum

(Liu & Hu, 1962)

ED4A07DA-DA96-5535-9639-43F66299F223

##### Distribution

Vietnam, China, India, Myanmar, Laos, Thailand, Cambodia.

##### Notes

Distribution in Tay Yen Tu Nature Reserve, Bac Giang Province (Hecht et al. 2013 and Poyarkov et al. 2015).

#### Theloderma corticale

(Boulenger, 1903)

55E7EFBE-0C62-5A44-91F3-3FA67FDE0CEE

##### Distribution

Vietnam, China, Laos.

##### Notes

Distribution in Tay Yen Tu Nature Reserve, Bac Giang Province (Hecht et al. 2013).

#### Theloderma lateriticum

Bain, Nguyen & Doan, 2009

DF69217F-9676-54CE-8F5C-62DC07F18DCB

##### Distribution

Vietnam, China, Laos.

##### Notes

Distribution in Tay Yen Tu Nature Reserve, Bac Giang Province (Hecht et al. 2013).

#### Zhangixalus pachyproctus

Yu, Hui, Hou, Wu, Rao & Yang, 2019

79D033FD-CABF-521C-B679-8EC0350AB078

##### Distribution

Vietnam, China, Laos.

##### Notes

Distribution in Tay Yen Tu Nature Reserve, Bac Giang Province (Hecht et al. 2013 and Yu et al. 2019).

#### Caudata

E4C8E8D7-3CC7-52B6-9BD8-C665AA4480F6

#### Salamandridae

139A4EF9-3A99-5652-9023-DCB00F6FE91F

#### Tylototriton vietnamensis

Böhme, Schöttler, Nguyen & Köhler, 2005

87BA2510-399D-5307-ABB1-815CF03A287D

##### Distribution

Vietnam, Laos.

##### Notes

Distribution in Tay Yen Tu Nature Reserve, Bac Giang Province (Hecht et al. 2013).

#### Gymnophiona

FF6B278C-1CFF-556D-9BC6-B61B6F80EC8E

#### Ichthyophiidae

2D69469B-5195-5CF7-8ED3-488560F27E5B

#### Ichthyophis kohtaoensis

Taylor, 1960

B54A0A2D-197A-533F-8C31-E58DEA764796

##### Distribution

Vietnam, China, Laos, Thailand, Cambodia.

##### Notes

Distribution in Tay Yen Tu Nature Reserve, Bac Giang Province (Hecht et al. 2013 and Nishikawa et al. 2021).

#### Reptilia

3C189AE8-2EEC-5DAB-83B3-B44583E59905

#### Squamata

E754ABFD-9B60-5198-BF2B-FF5EFF835060

#### Agamidae

AB78E0C3-1E5C-5BE3-9E92-139F72D72237

#### Acanthosaura lepidogaster

(Cuvier, 1829)

EE991A50-1803-5E7C-B306-BB75D800DE40

##### Distribution

Vietnam, China, Myanmar, Laos, Thailand, Cambodia.

##### Notes

Distribution in Tay Yen Tu Nature Reserve, Bac Giang Province (Hecht et al. 2013).

#### Draco maculatus

(Gray, 1845)

671181C1-343F-5392-8C6A-DFD2C50497C5

##### Distribution

Vietnam, India, China, Myanmar, Laos, Thailand, Cambodia, Malaysia.

##### Notes

Distribution in Tay Yen Tu Nature Reserve, Bac Giang Province (Hecht et al. 2013).

#### Physignathus cocincinus

(Cuvier, 1829)

F5CE5C4D-C288-515C-838F-85D5E0291D7C

##### Distribution

Vietnam, China, Myanmar, Laos, Thailand, Cambodia.

##### Notes

Distribution in Tay Yen Tu Nature Reserve, Bac Giang Province (Hecht et al. 2013).

#### Gekkonidae

7B286E89-1116-59A5-831B-3A30A994B247

#### Gekko palmatus

Boulenger, 1907

F8172AD1-959F-5247-85B0-1347FC770812

##### Distribution

Vietnam, China.

##### Notes

Distribution in Tay Yen Tu Nature Reserve, Bac Giang Province (Hecht et al. 2013).

#### Hemidactylus frenatus

Schlegel, 1836

B560BB75-9485-567D-8166-C3FBB8740C14

##### Distribution

Vietnam, worldwide in tropical and subtropical regions.

##### Notes

Distribution in Tay Yen Tu Nature Reserve, Bac Giang Province (Hecht et al. 2013).

#### Eublepharidae

CCB3DD79-071E-5536-B3DD-C5DC1D7ADBBC

#### Goniurosaurus lichtenfelderi

Mocquard, 1897

D8799E79-9F65-511E-8A19-4105CED0D134

##### Distribution

Vietnam, China.

##### Notes

Distribution in Tay Yen Tu Nature Reserve, Bac Giang Province (Hecht et al. 2013).

#### Scincidae

47497111-1923-546B-9341-637E7D4DC650

#### Ateuchosaurus chinensis

Gray, 1845

80E1239A-FD00-51BE-85CC-1B52608C087E

##### Distribution

Vietnam, China.

##### Notes

Distribution in Tay Yen Tu Nature Reserve, Bac Giang Province (Hecht et al. 2013).

#### Eutropis longicaudatus

(Hallowell, 1857)

6AD6C899-19A9-55DD-888D-30C0BC8A76A6

##### Distribution

Vietnam, China, Laos, Thailand, Cambodia, Malaysia.

##### Notes

Distribution in Tay Yen Tu Nature Reserve, Bac Giang Province (Hecht et al. 2013).

#### Eutropis multifasciatus

(Kuhl, 1820)

AEDC343D-71F5-59D6-B013-92EAA61FD360

##### Distribution

Vietnam, India, China, Taiwan, Myanmar, Laos, Thailand, Cambodia, Malaysia, Indonesia, Philippines, New Guinea.

##### Notes

Distribution in Tay Yen Tu Nature Reserve, Bac Giang Province (Hecht et al. 2013).

#### Sphenomorphus cryptotis

Darevsky, Orlov & Ho, 2004

CC4692D0-161A-599A-8292-EB354399400E

##### Distribution

Vietnam.

##### Notes

Distribution in Tay Yen Tu Nature Reserve, Bac Giang Province (Hecht et al. 2013).

#### Sphenomorphus indicus

(Gray, 1853)

DB1B61B9-890B-58CF-906D-71E4E355E57F

##### Distribution

Vietnam, India, Bhutan, China, including Taiwan, Myanmar, Laos, Thailand, Cambodia, Malaysia, Indonesia.

##### Notes

Distribution in Tay Yen Tu Nature Reserve, Bac Giang Province (Hecht et al. 2013).

#### Sphenomorphus incognitus

(Thompson, 1912)

EC096E1A-3603-523C-A3EB-3F40C92D754A

##### Distribution

Vietnam, China.

##### Notes

Distribution in Tay Yen Tu Nature Reserve, Bac Giang Province (Hecht et al. 2013 and Nguyen et al. 2012).

#### Sphenomorphus tonkinensis

Nguyen, Schmitz, Nguyen, Orlov, Böhme & Ziegler, 2011

EFCE11F1-9FDA-53EB-BF17-301C30B0EB5E

##### Distribution

Vietnam.

##### Notes

Distribution in Tay Yen Tu Nature Reserve, Bac Giang Province (Hecht et al. 2013).

#### Plestiodon tamdaoensis

(Bourret, 1937)

56166126-329E-5AB1-881E-7D935FD0E398

##### Distribution

Vietnam, China.

##### Notes

Distribution in Tay Yen Tu Nature Reserve, Bac Giang Province (Hecht et al. 2013).

#### Tropidophorus hainanus

Smith, 1923

27BABBD7-0E65-5A81-ACB5-8D9FAD5882FB

##### Distribution

Vietnam, China.

##### Notes

Distribution in Tay Yen Tu Nature Reserve, Bac Giang Province (Hecht et al. 2013).

#### Tropidophorus sinicus

Boettger, 1886

B433159E-5646-5AC7-B809-80F47BE65BA0

##### Distribution

Vietnam, China.

##### Notes

Distribution in Tay Yen Tu Nature Reserve, Bac Giang Province (Hecht et al. 2013).

#### Shinisauridae

7825EF72-DBB5-5C23-A2FD-44D5F2EF3FE8

#### Shinisaurus crocodilurus

Ahl, 1930

23FC82EA-01B3-590C-9D52-21BCAEB0579C

##### Distribution

Vietnam, China.

##### Notes

Distribution in Tay Yen Tu Nature Reserve, Bac Giang Province (Hecht et al. 2013).

#### Lacertidae

C1549037-4E4B-5CB7-84BE-E30D9F4DAAA7

#### Takydromus kuehnei

Van Denburgh, 1909

7540483B-1097-57E4-BD99-7DF42F263BCF

##### Distribution

Vietnam, China.

##### Notes

Distribution in Tay Yen Tu Nature Reserve, Bac Giang Province (Hecht et al. 2013).

#### Takydromus sexlineatus

Daudin, 1802

83FFB14F-62B0-52DD-9DCB-AFA0316CBA14

##### Distribution

Vietnam, India, China, Myanmar, Laos, Thailand, Cambodia, Malaysia, Indonesia.

##### Notes

First record for Bac Giang Province.

#### Colubridae

601F0A1F-E8DC-5DC4-99A7-B65847CBE77D

#### Ahaetulla prasina

(Boie, 1827)

CD68D2CF-6DAE-54A2-82D2-AE486148700C

##### Distribution

India, Bhutan, Myanmar, Cambodia, Laos, Vietnam, Malaysia, China, Indonesia, the Philippines.

##### Notes

Distribution in Tay Yen Tu Nature Reserve, Bac Giang Province (Hecht et al. 2013).

#### Amphiesmoides ornaticeps

(Werner, 1924)

DAABE631-2A73-574D-AB42-4A7E7F71D72C

##### Distribution

Vietnam, China.

##### Notes

Distribution in Tay Yen Tu Nature Reserve, Bac Giang Province (Hecht et al. 2013).

#### Boiga kraepelini

Stejneger, 1902

83977C50-51FD-59E5-BF8B-19E7D7CD412D

##### Distribution

China, Vietnam, Laos.

##### Notes

Distribution in Tay Yen Tu Nature Reserve, Bac Giang Province (Hecht et al. 2013 and Ziegler et al. 2010).

#### Calamaria pavimentata

Duméril, Bibron & Duméril, 1854

421F5F81-0A14-5C49-A634-58B8023358FC

##### Distribution

India, Malaysia, Thailand, Laos, Vietnam, Cambodia, Myanmar, China.

##### Notes

Distribution in Tay Yen Tu Nature Reserve, Bac Giang Province (Hecht et al. 2013).

#### Calamaria septentrionalis

Boulenger, 1890

F65B360A-AF95-5A87-A686-1EE6274E79A1

##### Distribution

China, Vietnam.

##### Notes

Distribution in Tay Yen Tu Nature Reserve, Bac Giang Province (Hecht et al. 2013).

#### Coelognathus radiatus

(Boie, 1827)

85C8FFF6-2457-50A0-B467-9A182A67D12D

##### Distribution

Indonesia, Malaysia, Borneo, Burma, Thailand, Laos, Cambodia, Vietnam, India, Banglagesh, Nepal, Bhutan, China.

##### Notes

Distribution in Tay Yen Tu Nature Reserve, Bac Giang Province (Nguyen et al. 2009).

#### Gonyosoma coeruleum

Liu, Hou, Lwin, Wang & Rao, 2021

F867667A-DB93-562A-873A-0A669D8F3865

##### Distribution

China, Myanmar, Malaysia, Thailand, Vietnam.

##### Notes

Distribution in Tay Yen Tu Nature Reserve, Bac Giang Province (Hecht et al. 2013, Liu et al. 2021).

#### Gonyosoma boulengeri

(Mocquard, 1897)

ADC9DF07-185A-545A-B85B-27F7F7F13742

##### Distribution

Vietnam, China.

##### Notes

Distribution in Tay Yen Tu Nature Reserve, Bac Giang Province (Hecht et al. 2013).

#### Hypsiscopus murphyi

Bernstein, Voris, Stuart, Phimmachak, Seateun, Sivongxay, Neang, Karns, Andrews, Osterhage, Phipps & Ruane, 2022

8B8D80BA-4B37-5F40-9E05-15DB7F02F86B

##### Distribution

Cambodia, China, Laos, Thailand, Vietnam.

##### Notes

Distribution in Tay Yen Tu Nature Reserve, Bac Giang Province (Nguyen et al. 2009, Murphy and Voris 2014 and Bernstein et al. 2022).

#### Lycodon futsingensis

(Pope, 1928)

6FCE9108-A99B-5DDD-AF21-1BAE235BEB94

##### Distribution

China, Vietnam, Laos.

##### Notes

Distribution in Tay Yen Tu Nature Reserve, Bac Giang Province (Hecht et al. 2013).

#### Lycodon flavozonatus

Pope (1928)

321EAF07-EE0A-59FE-8D96-B32A0E368FB8

##### Distribution

Vietnam, Laos, China.

##### Notes

Distribution in Tay Yen Tu Nature Reserve, Bac Giang Province (Hecht et al. 2013 and Li et al. 2025).

#### Oligodon chinensis

(Günther, 1888)

48917DE8-4558-597A-BDE5-07656AB5DD73

##### Distribution

Vietnam, China.

##### Notes

Distribution in Tay Yen Tu Nature Reserve, Bac Giang Province (Hecht et al. 2013).

#### Opisthotropis lateralis

Boulenger, 1903

9F857E28-42BF-5940-9B80-EE911FD8EA1A

##### Distribution

Vietnam, China.

##### Notes

Distribution in Tay Yen Tu Nature Reserve, Bac Giang Province (Hecht et al. 2013).

#### Opisthotropis voquyi

Ziegler, David, Ziegler, Pham, Nguyen & Le, 2018

8F11ADA4-9BA8-5AF0-AC7D-13AC7787D1F1

##### Distribution

Vietnam.

##### Notes

Distribution in Tay Yen Tu Nature Reserve, Bac Giang Province (Ziegler et al. 2018).

#### Ptyas multicincta

(Roux, 1907)

08B936E8-23D3-56F0-B198-E5734936C87F

##### Distribution

China, Laos, Vietnam, Thailand.

##### Notes

Distribution in Tay Yen Tu Nature Reserve, Bac Giang Province (Hecht et al. 2013 and Figueroa et al. 2016).

#### Rhabdophis helleri

(Schmidt, 1925)

26B4C97B-52D0-55DD-B1A9-B1B329BEC514

##### Distribution

Indonesia, Malaysia, Vietnam.

##### Notes

Distribution in Tay Yen Tu Nature Reserve, Bac Giang Province (Hecht et al. 2013 and Liu et al. 2020).

#### Sibynophis chinensis

(Günther, 1889)

8DBF572B-1377-5AFB-83BF-222851050D0F

##### Distribution

Vietnam, China.

##### Notes

Distribution in Tay Yen Tu Nature Reserve, Bac Giang Province (Hecht et al. 2013).

#### Trimerodytes aequifasciatus

(Barbour, 1908)

E60E38F2-6795-59F4-9531-DD69A079C769

##### Distribution

China, Vietnam, Laos.

##### Notes

Distribution in Tay Yen Tu Nature Reserve, Bac Giang Province (Hecht et al. 2013 and Ren et al. 2019).

#### Trimerodytes percarinatus

(Boulenger, 1899)

A76351FE-766B-5E5B-8448-67AD034A86B8

##### Distribution

Myanmar, Thailand, Vietnam, India, China.

##### Notes

Distribution in Tay Yen Tu Nature Reserve, Bac Giang Province (Hecht et al. 2013 and Ren et al. 2019).

#### Psammodynastidae

3343AE51-3861-5FBE-913A-8A4CFEE27529

#### Psammodynastes pulverulentus

(Boie, 1827)

D041A27F-A023-5603-BCD6-7AF6DD2F7299

##### Distribution

Bangladesh, Burma, Cambodia, China, India, Bhutan, Indonesia, Laos, the Philippines, Thailand, Vietnam, Malaysia.

##### Notes

Distribution in Tay Yen Tu Nature Reserve, Bac Giang Province (Hecht et al. 2013).

#### Pareidae

F38A71F7-3987-5A6D-A1FE-76AF5C760E77

#### Pareas hamptoni

(Boulenger, 1905)

3CD3B3BA-526C-5A44-9208-5C8410F2674E

##### Distribution

Myanmar, Vietnam, China.

##### Notes

Distribution in Tay Yen Tu Nature Reserve, Bac Giang Province (Hecht et al. 2013).

#### Pareas margaritophorus

(Jan, 1866)

75526A45-E6D1-5B09-B9B6-0D1D13C76992

##### Distribution

Malaysia, Indonesia, Thailand, Laos, Cambodia, Vietnam, China.

##### Notes

Distribution in Tay Yen Tu Nature Reserve, Bac Giang Province (Hecht et al. 2013).

#### Elapidae

7120A7C9-9E03-520D-B69D-E7634C53D457

#### Bungarus fasciatus

(Schneider, 1801)

6372D453-65A4-5EDA-A67F-13D4F4709682

##### Distribution

Bangladesh, Burma, Cambodia, China, India, Bhutan, Nepal, Indonesia, Laos, Malaysia, Singapore, Thailand, Vietnam.

##### Notes

Distribution in Tay Yen Tu Nature Reserve, Bac Giang Province (Hecht et al. 2013).

#### Sinomicrurus macclellandi

(Reinhardt, 1844)

1808EB88-01BD-5B0C-8DE2-C17AEF0A8C7E

##### Distribution

India, Nepal, Bangladesh, Bhutan, Thailand, Vietnam, China, India, Nepal.

##### Notes

First record for Bac Giang Province.

#### Viperidae

A9B21291-1E26-541E-94B1-C1D771A69F64

#### Trimeresurus stejnegeri

Schmint, 1925

1CA21604-D250-5A59-A7FC-3483F0C2AF64

##### Distribution

China, India, Myanmar, Vietnam, Laos.

##### Notes

Distribution in Tay Yen Tu Nature Reserve, Bac Giang Province (Hecht et al. 2013).

#### Typhlopidae

71A6815D-BC39-5ADB-BF39-19E23AD07F29

#### Indotyphlops braminus

(Daudin, 1803)

B2EB303B-3A0C-550C-807F-69D8BEB5970C

##### Distribution

Vietnam, Zanzibar, Tanzania, Mozambique, Somalia, Cameroon, Benin, Togo, Ivory Coast, Senegal, Gabon, Madagascar, Comores, Nossi Be, Mauritius, Pakistan, India, Sri Lanka, Nepal, Bangladesh, Bhutan, China, Myanmar, Laos, Thailand, Cambodia, Malaysia, Singapore, Indonesia, the Philippines, Japan, Melanesia, Micronesia, Australia, New Caledonia, Solomon Islands, Vanuatu. Additionally in western Asia (Saudi Arabia) and America (Guatemala, Mexico, USA).

##### Notes

Distribution in Tay Yen Tu Nature Reserve, Bac Giang Province (Hecht et al. 2013)

#### Xenodermidae

CC5F75FF-3B04-5BE0-B1E8-F341139D8D59

#### Achalinus emilyae

Ziegler, Nguyen, Pham, Nguyen, Pham, Van Schingen, Nguyen & Le, 2019

FDB4CE0D-66DA-5DE8-8E48-90F2E0F52DE9

##### Distribution

Vietnam, China.

##### Notes

Distribution in Tay Yen Tu Nature Reserve, Bac Giang Province (Ziegler et al. 2019).

#### Xenopeltidae

DFB983B3-A390-5981-A31F-80FE6521691E

#### Xenopeltis hainanensis

Hu & Zhao, 1972

3CCCF09D-844F-520C-A824-B4CFA3B43A07

##### Distribution

China, Vietnam.

##### Notes

First record for Bac Giang Province.

#### Testudines

F25F7871-9B36-5E11-A04F-CAE8B08833BB

#### Geoemydidae

CC01113E-9689-5E2D-B526-A2369C3CB27D

#### Geoemyda spengleri

(Gmelin, 1789)

77938207-1905-5A52-B7F5-0406A3023D32

##### Distribution

China, Vietnam, Laos.

##### Notes

Distribution in Tay Yen Tu Nature Reserve, Bac Giang Province (Pham et al. 2018).

#### Cuora mouhotii

(Gray, 1862)

F8ECDBA1-7D2B-5675-BD5C-9F6F25C957B7

##### Distribution

China, Vietnam, Thailand, Myanmar, India, Bhutan, Laos.

##### Notes

Distribution in Tay Yen Tu Nature Reserve, Bac Giang Province (Pham et al. 2018).

## Discussion

At present, a total of 44 species of amphibians belonging to 28 genera (eight families, three orders) and 49 species of reptiles belonging to 37 genera (15 families, two orders) are known from Tay Yen Tu Nature Reserve. It is noted that the taxonomic assignment of some species have been changed. For example, *Xenophrysmajor* (Boulenger, 1908) was re-identified as *X.maosonensis* (Bourret, 1937) (Mahony et al. 2018), *Amolopsricketti* (Boulenger, 1899) was re-assigned to *A.shihaitaoi* Wang, Li, Du, Hou & Yu, 2022 (Wang et al. 2022), *Hylarananigrovittata* (Blyth, 1856) to *H.annamitica* (Sheridan & Stuart, 2018) (Sheridan and Stuart 2018), *Odorranabacboensis* (Bain, Lathrop, Murphy, Orlov & Ho, 2003) to *O.fengkaiensis* Wang, Lau, Yang, Chen, Liu, Pang & Liu, 2015 (Pham et al. 2020), *Rhacophorusrhodopus* Liu & Hu, 1960 to *R.napoensis* Li, Liu, Yu & Sun, 2022(Li et al. 2022 and Nguyen et al. 2024),*Thelodermaasperum* (Boulenger, 1886) to *T.albopunctatum* (Liu and Hu, 1962) (Poyarkov et al. 2015), *Rhacophorusmaximus* (Rao, Wilkinson & Liu, 2006) to *Zhangixaluspachyproctus* Yu, Hui, Hou, Wu, Rao & Yang, 2019 (Yu et al. 2019), *Ichthyophisbannanicus* Yang, 1984 to *I.kohtaoensis* Taylor, 1960 (Nishikawa et al. 2021), *Lycodonmeridionalis* (Bourret, 1935) to *L.flavozonatus* Pope (1928) (Li et al. 2025), *Hypsiscopusplumbea* (Boie, 1827) to *H.murphyi* Bernstein, Voris, Stuart, Phimmachak, Seateun, Sivongxay, Neang, Karns, Andrews, Osterhage, Phipps & Ruane, 2022 (Bernstein et al. 2022), *Rhabdophissubminiatus* (Schlegel, 1837) to *R.helleri* (Schmidt, 1925) (Liu et al. 2020) and *Achalinusrufescens* Boulenger, 1888 to *A.emilyae* Ziegler, Nguyen, Pham, Nguyen, Pham, Van Schingen, Nguyen & Le, 2019 (Ziegler et al. 2019). Our new findings not only fill knowledge gaps of the herpetofaunal diversity, but also underline the importance of the Tay Yen Tu Nature Reserve in terms of biodiversity conservation in Vietnam.
